# Environmental and clinical data utility in pediatric asthma exacerbation risk prediction models

**DOI:** 10.1186/s12911-022-01847-0

**Published:** 2022-04-22

**Authors:** Jillian H. Hurst, Congwen Zhao, Haley P. Hostetler, Mohsen Ghiasi Gorveh, Jason E. Lang, Benjamin A. Goldstein

**Affiliations:** 1 Department of Pediatrics, Division of Infectious Diseases, Duke University School of Medicine, Durham, NC USA; 2 Department of Pediatrics, Children’s Health and Discovery Initiative, Duke University School of Medicine, Durham, NC USA; 3 Department of Biostatistics and Bioinformatics, Duke University School of Medicine, Durham, NC USA; 4 Department of Medicine, Division of Pulmonary, Allergy, and Critical Care Medicine, Duke University School of Medicine, Durham, NC USA; 5 Duke Clinical Research Institute, Duke University, Durham, NC USA; 6 Department of Pediatrics, Division of Pulmonary and Sleep Medicine, Duke University School of Medicine, Durham, NC USA

**Keywords:** Asthma, Pediatrics, Predictive modeling, Environmental data, Machine learning

## Abstract

**Background:**

Asthma exacerbations are triggered by a variety of clinical and environmental factors, but their relative impacts on exacerbation risk are unclear. There is a critical need to develop methods to identify children at high-risk for future exacerbation to allow targeted prevention measures. We sought to evaluate the utility of models using spatiotemporally resolved climatic data and individual electronic health records (EHR) in predicting pediatric asthma exacerbations.

**Methods:**

We extracted retrospective EHR data for 5982 children with asthma who had an encounter within the Duke University Health System between January 1, 2014 and December 31, 2019. EHR data were linked to spatially resolved environmental data, and temporally resolved climate, pollution, allergen, and influenza case data. We used xgBoost to build predictive models of asthma exacerbation over 30–180 day time horizons, and evaluated the contributions of different data types to model performance.

**Results:**

Models using readily available EHR data performed moderately well, as measured by the area under the receiver operating characteristic curve (AUC 0.730–0.742) over all three time horizons. Inclusion of spatial and temporal data did not significantly improve model performance. Generating a decision rule with a sensitivity of 70% produced a positive predictive value of 13.8% for 180 day outcomes but only 2.9% for 30 day outcomes.

**Conclusions:**

EHR data-based models perform moderately wellover a 30–180 day time horizon to identify children who would benefit from asthma exacerbation prevention measures. Due to the low rate of exacerbations, longer-term models are likely to be most clinically useful.

*Trial Registration*: Not applicable.

**Supplementary Information:**

The online version contains supplementary material available at 10.1186/s12911-022-01847-0.

## Introduction

Asthma is a chronic airway disease that affects over five million children in the United States [1]. While asthma can often be well controlled via medical therapy, including through the use of regular controller medications such as inhaled corticosteroids, exacerbations requiring emergency treatment are common. Over half of all children with asthma experience an exacerbation each year, with one in six visiting an emergency department and one in 20 requiring hospitalization for an asthma exacerbation [2, 3]. Importantly, asthma exacerbations comprise the majority of asthma-related healthcare costs, and there is a significant need to better identify children at high-risk for future exacerbations to allow targeted preventive interventions.

Asthma exacerbations are known to be triggered by a variety of clinical, environmental, and seasonal exposures; however, the interplay of these factors and their impacts on the risk of exacerbation are not well understood [4]. For example, children with asthma and seasonal allergies may experience an increase in exacerbation risk when pollen counts are high, whereas children without seasonal allergies will not be affected. Similarly, air pollution may have a significant impact on children who live in neighborhoods that are close to highways. Efforts to prevent asthma exacerbations have focused on evidence-based efforts such as patient/family education, asthma action plans, identification and remediation of environmental triggers, identification and treatment of contributing comorbidities such as atopic disease and obesity, and methods to improve asthma controller medication adherence. Given the significant heterogeneity in asthma presentations and risk factors, it is difficult to identify patients who are at greatest risk of exacerbations and who may benefit most from targeted interventions [5]. Moreover, the contribution of different types of exposures in predicting future asthma exacerbations has not been well-studied.

Over the past decade, electronic health record (EHR) systems, which contain detailed patient-level clinical data, have allowed the development and implementation of automated clinical decision support (CDS) tools [6]. Such tools have the potential to assist in the identification of patients at highest risk of poor outcomes in a variety of disease states, including asthma. To date, development of asthma exacerbation risk prediction models has focused on healthcare utilization and clinical characteristics [4]; however, the numerous factors that can affect exacerbation risk are not captured by most EHR systems, including housing and neighborhood characteristics and changes in common contributing exposures such as weather, outdoor allergens, and respiratory infections. There is a critical need to evaluate how such contextualizing information could potentially improve asthma CDS tools. The goal of this study was to evaluate the contribution of forms of data that are not typically available within the EHR (i.e., spatially and temporally resolved environmental data) to the performance of asthma exacerbation prediction models. Herein, we combined clinical data from a cohort of children with asthma with spatial and temporal environmental data to assess how well these different data sources contributed to the performance of risk prediction models for asthma exacerbations over different time horizons.

## Materials and methods

### Study population

**Fig. 1:**
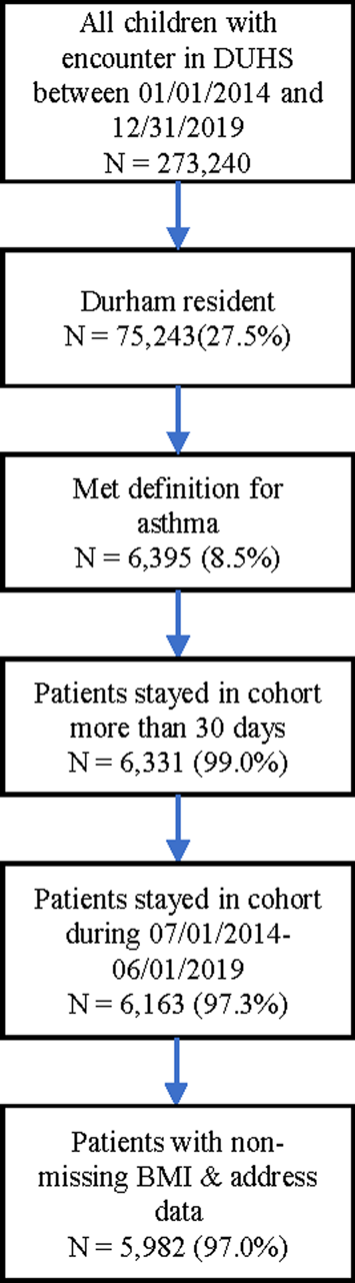
Consolidated standards of reporting trials diagram

The study was conducted using retrospective data from Duke University Health System (DUHS). DUHS consists of one tertiary care and two community-based hospitals, and a network of primary care and specialty clinics that have utilized a single EHR system since 2014. DUHS is the primary provider in Durham County, North Carolina, and we have internally estimated that ~ 85 percent of children in Durham County receive healthcare through DUHS [7]. We abstracted clinical data through Duke’s EHR-based Clinical Research Datamart from January 1, 2014, to December 31, 2019 [8]. We identified children (age 5–18), living in Durham County with asthma. We used a previously validated definition that has a positive predictive value of 97% [9]. As previously described, we classified children as having asthma if they met one of the following sets of criteria: (1) two or more outpatient or emergency health care encounters associated with an International Classification of Diseases, Ninth/Tenth Revision (ICD-9/ICD-10) code for asthma (Additional file 1: Table S1) and an active prescription for one or more medications for asthma (Additional file 1: Table S2); (2) at least one hospital encounter associated with an ICD-9/ICD-10 for asthma and an active prescription for one of more medications for asthma; or (3) a problem list entry with an asthma-related ICD-9/ICD-10 code and an active prescription for one or more medications for asthma (Fig. 1). We identified 6395 children who met the study criteria for asthma; of these, 6163 were in the cohort for the full study period. Of these patients, 181 were missing either address or BMI data during the study period and were therefore excluded from further analysis.

Patient person-time was calculated from time of positive asthma identification until censoring. Censoring was based on aging out of the cohort (≥ 18 years), an indicated address outside of Durham County, or at the last known encounter. Additionally, we applied a six-month burn-in and burn-out period to ensure data reliability, resulting in a total of five years of data (July 1, 2014 – June 30, 2019).

### Outcome of interest

The primary outcome of interest was an asthma-related exacerbation, which was defined as any encounter with an asthma-related ICD9 or -10 code and a prescription for a systemic steroid (see Additional file 1: Table S2). We considered four different types of exacerbations based on severity (listed in decreasing severity): (1) inpatient encounters lasting more than 24 h, (2) emergency department and hospital encounters lasting less than 24 h, (3) urgent care visits, and (4) outpatient (including telephone-based) encounters.

### Predictor variables

Clinical predictors were abstracted from EHR data and were updated on a person-month basis. We abstracted clinical and socio-demographic information on each child from the EHR, including sex, age, race, insurance type (public, private, self-pay), comorbidities (atopy, obesity), medication prescriptions. Each participant was categorized based on type of prescribed asthma controller plan (i.e., only rescue medications, only inhaled corticosteroids or only leukotriene receptor antagonists, or other controller medications). Patient service utilization history (including asthma-related encounters and encounters for other indications) was separated into three categories: ambulatory visits (i.e., any outpatient care, including specialist care and sick visits at both urgent care centers and primary care providers, regardless of associated diagnoses), emergency department encounters, and inpatient admissions. Well child visits were considered as a separate category, as prior work has demonstrated a beneficial effect of these visits among children with asthma [10].

### Spatial data

Neighborhood-level environmental data were derived based on patient address. We used the latitude–longitude of patient time-resolved address to geocode each patient. We identified each child’s zip code of residence and linked data from the American Community Survey to calculate the Agency for Healthcare Research and Quality (AHRQ) socioeconomic status (SES) index, generating a score between 0 and 100, with higher scores indicative of greater deprivation [11]. We additionally calculated distance to major roadways with speed limits greater than 55 MPH as described previously, and distance to parks, and tree cover for the census block associated with each address [12]. Briefly, we used ArcGIS to calculate straight-line distance to roadways for each geocoded address within our dataset.

### Temporal data

We downloaded daily climate data on daily average temperature, total precipitation, maximum wind speed from the National Centers for Ennvironmental Information (https://www.ncdc.noaa.gov/). Air quality data, including the maximum sulfur dioxide (SO_2_) reading and average particulate matter 2.5 (PM2.5) concentration were downloaded from the US Environmental Protection Agency (https://www.epa.gov/outdoor-air-quality-data/download-daily-data). Pollen counts for trees weeds and grasses were downloaded from the North Carolina Department of Environmental Quality Pollen Monitoring program (https://deq.nc.gov/about/divisions/air-quality/air-quality-monitoring/pollen-monitoring). Pollen data were log transformed to normalize the data, which is required for use in linear-based modeling methods such as LASSO. Pollen data were not available in winter months, as the local monitoring station does not take readings during this period due to the very low levels of pollen during winter; thus, these periods were imputed to have pollen counts of zero. We also calculated seasonal influenza burden by abstracting the daily number of influenza tests performed at the institution in that month. We also included index month from when the prediction would be made.

### Data formatting

Our goal was to develop a CDS tool that identifies children on a monthly basis at greatest risk for asthma exacerbation. As such, we organized the data into patient month format, creating a row for each month a patient was eligible to be included in the study, and updating all time varying factors. Time varying factors included: address (using the last known value in the prior month); insurance (using the last known value in the prior month); number of healthcare encounters (outpatient, emergency, and inpatient), regardless of cause, in the previous 30 and 365 days; an indicator for whether a child had a well-child visit in the previous year; and the average of each of the temporal factors in the prior month. To build a predictive model, we generated three outcomes for each patient-month: whether a patient had an exacerbation in the forthcoming 30-, 90- and 180- days.

### Statistical analysis

We divided the data at the patient level into training and testing sets in a 67%/33% ratio. We used LASSO, Random Forests, and xgBoost to build our predictive models. LASSO, or Least Absolute Shrinkage and Selection Operator, is a linear regression-based model that uses shrinkage and L1 regularization to produce sparse models. The glmnet package in R was used was used to create the LASSO models [13]. Random Forests is a tree-based machine learning algorithm that can handle disparate data types and model complex effects (i.e., non-linearities and interactions). The ranger package in R was used to create Random Forest models [14]. xgBoost, or Extreme Gradient Boosting, is a gradient-boosted decision tree machine learning library that utilizes iterative learning to optimize prediction. The gbm package in R was used to develop xgBoost models [15]. Training data were used to optimize each algorithm, and model tuning parameters were chosen via internal cross-validation. For LASSO, we optimized the lambda (shrinkage) parameter. For Random Forests we optimized the “mtry” (variable to select) parameter, fixing the algorithm at 4000 trees. For xgBoost, we set the number of splits at two and the learning rate at 0.01 and learned an early stopping rule for the number of trees. We initially built 15 different primary models, using three different time horizons with 5 different sets of predictors. We first used all of the predictor variables to train a model to predict 30-, 90-, and 180-day risk of exacerbation. Next, we separated the predictor variables based on whether they were clinical, neighborhood or environmental factors, fitting separate models for each predictor group (see Additional file 1: Table S3 for a description of the factors included in each model). Finally, we considered a simpler, parsimonious model that includes data readily available to both patients/families and clinicians: age, sex, race, presence of either atopy or obesity, and current medications. The ROCR package in R was used to evaluate model performance and to compare results across models [16]. We used the test data to calculate the area under the receiver operator characteristic (AUROC). We used the bootstrap, resampling at the patient level, to calculate 95% confidence intervals. We compared the performances of the different models by calculating the delta AUROC and a bootstrap for 95% confidence intervals. Finally, we assessed the impact of decision making by calculating the Precision-Recall Curve and evaluated the sensitivity and positive predictive value (PPV) at different cut-points. All analyses were performed in R 4.1.0 [17]. This work was approved by the Duke University Health System IRB.

## Results

### Patient characteristics

We identified 5982 children with a total of 17,907.56 patient-years (Fig. 1, Table 1). The patient population had slightly more male than female patients (56%). The majority of patients in the cohort were listed as non-Hispanic Black (58.1%); 12.4% of patients were of Hispanic ethnicity; 20.2% of patients were identified as non-Hispanic white, and 9.4% of patients were of unknown or other race/ethnicity. A majority of patients in the cohort had a history of atopy (62%) and allergic rhinitis (56%) (Table 1).

**Table 1:** Characteristics of the study population

Characteristics	N or Median	IQR or %
Age	8.7	(6.5, 12.5)
Sex	N	%
Female	2792	43.7%
Male	3603	56.3%%
Race/Ethnicity	N	%
Hispanic	790	12.4%
Non-Hispanic Black	3713	58.1%
Non-Hispanic White	1289	20.2%
Other/Unknown	603	9.4%
Comorbidities*	N	%
History of any atopic disease	3929	61.4%%
Allergic rhinitis and conjunctivitis	3485	54.5%
Food allergy	325	5.1%
Eczema	992	15.5%
Obesity	1489	25.4%

Exacerbation rate	Rate (100 person-years)
All exacerbations	27.65
Hospital encounters > 24 h	2.11
ED and hospital encounters < 24 h	6.94
Urgent care encounters	6.25
All other outpatient encounters	12.35

**Fig. 2:**
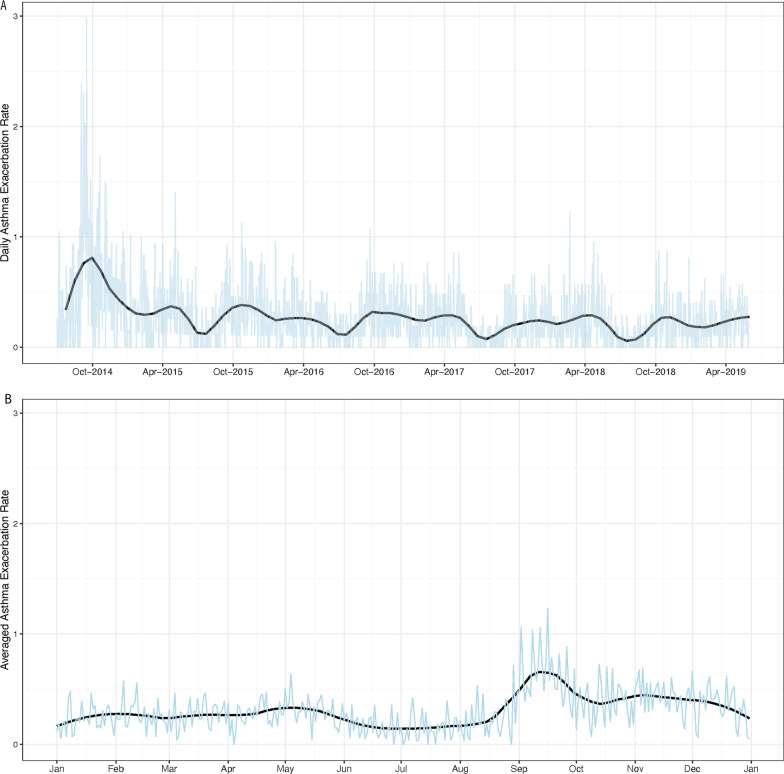
Asthma exacerbation rates across the study period. **A** The number of asthma exacerbations per month during the study period. **B** The average number of asthma exacerbations observed in each calendar month during the study period

There were 5045 exacerbations documented in our dataset, with an average of 0.27 exacerbations per patient year; 37% of patients had at least one asthma exacerbation during the study period. We evaluated the seasonal variability of asthma exacerbation incidence during the observation period (Fig. 2), and identified September as the month with the greatest average number of exacerbations, as has been documented previously [18].

### Performance of predictive models

We created person-month models using LASSO, Random Forest, and xgBoost models that used all available data (Table 2, “Overall”), including clinical, spatial, and temporal factors. The predicted event rate and AUC for the three time horizons (30-, 90-, and 180-days) are shown in Table 2. Model performance was better for near-term than for long-term outcomes for all modeling approaches. Performance of models developed using Gradient Boosting was nominally better than models using either LASSO or Random Forests. We evaluated the relative contributions of temporal, spatial, and clinical factors on model performance for predicting exacerbations. We found that clinical factors drove most of the model performance, regardless of modeling approach, with the temporal factors having reduced predictive value and spatial factors having minimal predictive value.

**Table 2:** AUC for predicting asthma exacerbation over different time horizons and variable sets using different modeling methods

Outcome	Predicted Event Rate	Overall model		Temporal factors		Clinical factors		Spatial factors		Parsimonious model	
		AUC	95% CI	AUC	95% CI	AUC	95% CI	AUC	95% CI	AUC	95% CI
AUC of Person-Month LASSO Models											
Exacerbation in 30 days	0.015	0.753	(0.732, 0.773)	0.600	(0.594, 0.616)	0.734	(0.713, 0.756)	0.502	(0.475, 0.535)	0.667	(0.638, 0.694)
Exacerbation in 90 days	0.043	0.740	(0.718, 0.761)	0.576	(0.562, 0.591)	0.732	(0.709, 0.753)	0.492	(0.462, 0.521)	0.643	(0.616, 0.670)
Exacerbation in 180 days	0.077	0.732	(0.710, 0.753)	0.547	(0.533, 0.563)	0.729	(0.706, 0.751)	0.492	(0.463, 0.520)	0.645	(0.618, 0.672)
AUC of Person-Month Random Forest Survival Model**s**											
Exacerbation in 30 days	0.015	0.757	(0.736, 0.777)	0.608	(0.602, 0.624)	0.741	(0.720, 0.763)	0.502	(0.470, 0.535)	0.672	(0.647, 0.697)
Exacerbation in 90 days	0.043	0.747	(0.726, 0.750)	0.582	(0.567, 0.599)	0.738	(0.717, 0.758)	0.498	(0.471, 0.524)	0.644	(0.619, 0.671)
Exacerbation in 180 days	0.077	0.729	(0.707, 0.750)	0.557	0.539, 0.577)	0.725	(0.703, 0.746)	0.541	(0.512, 0.570)	0.648	(0.623, 0.673)
AUC of Person-Month Gradient Boosting (xgBoost) Models											
Exacerbation in 30 days	0.015	0.761	(0.742, 0.781)	0.607	(0.601, 0.623)	0.742	(0.721, 0.763)	0.501	(0.469, 0.534)	0.664	(0.637, 0.688)
Exacerbation in 90 days	0.043	0.752	(0.730, 0.771)	0.581	(0.566, 0.597)	0.744	(0.722, 0.763)	0.488	(0.459, 0.517)	0.639	(0.613, 0.665)
Exacerbation in 180 days	0.077	0.739	(0.717, 0.760)	0.557	(0.538, 0.576)	0.730	(0.708, 0.752)	0.503	(0.474, 0.531)	0.640	(0.614, 0.665)

### Evaluation of the contribution of different types of data to model performance

To better understand the opportunity to create a model for which patients and/or their parents could readily provide the necessary information, we constructed a parsimonious model that uses basic demographics (age, race, ethnicity), comorbidities (atopy and obesity), and currently prescribed asthma medications. Below, we highlight the results of the xgBoost model, as its performance was nominally better than either Random Forest or LASSO. The parsimonious model (AUC = 0.664 for the 30-day time horizon) did not perform as well as the overall model (AUC = 0.761 for the 30-day time horizon) or the clinical factors-based model (AUC = 0.742 for the 30-day time horizon) (Table 2). When comparing the performance of the xgBoost model using all data elements to the one using only clinical data, we found the full model was nominally better for near term outcomes, and not any better for the longer 180-day outcome (Table 3). Conversely, when comparing the clinical model to the parsimonious model, we found that the performance of the clinical model was significantly better for all time horizons.

**Table 3:** Comparison of the overall, clinical, and parsimonious models created with different modeling methods

Outcome	AUC			*P* value	
	Overall model	Clinical factors	Parsimonious model	Overall model vs. Clinical factors	Clinical factors vs. Parsimonious model
Comparison of the overall, clinical, and parsimonious models of LASSO models					
Exacerbation in 30 days	0.753	0.734	0.667	< 0.001	< 0.001
Exacerbation in 90 days	0.740	0.732	0.643	< 0.001	< 0.001
Exacerbation in 180 days	0.732	0.729	0.645	0.026	< 0.001
Comparison of the overall, clinical, and parsimonious models of Random Forest Survival models					
Exacerbation in 30 days	0.757	0.741	0.672	< 0.001	< 0.001
Exacerbation in 90 days	0.747	0.738	0.644	< 0.001	< 0.001
Exacerbation in 180 days	0.729	0.725	0.648	0.019	< 0.001
Comparison of the overall, clinical, and parsimonious models of Gradient Boosting (xgBoost) models					
Exacerbation in 30 days	0.761	0.742	0.664	< 0.001	< 0.001
Exacerbation in 90 days	0.752	0.744	0.639	< 0.001	< 0.001
Exacerbation in 180 days	0.739	0.730	0.640	< 0.001	< 0.001

### Assessment of overall model sensitivity and positive predictive value

**Fig. 3:**
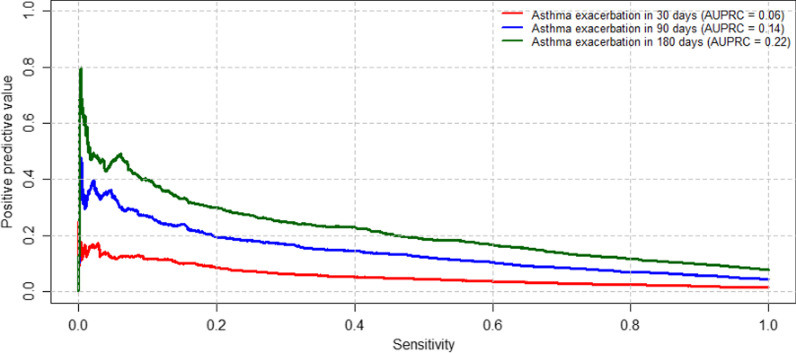
The relationship between the sensitivity and positive predictive value over three different time horizons. A Precision-Recall Curve was used to evaluate the sensitivity and positive predictive value (PPV) at different cut-points using a model based on clinical factors and patient characteristics (the “Clinical Factors” model)

Finally, we assessed the performance of a decision rule to guide clinical decision support using each of the models based on clinical factors (Fig. 3). For the 30-day time horizon, if we desire a sensitivity of ~ 70%, we would only have a PPV of ~ 2.9% using a xgBoost model. Conversely, if we used the 180-day time horizon, we would have a PPV of ~ 13.8%. Similarly, if we wanted a PPV of ~ 15% we would have a sensitivity of 66.2% from the 180-day time horizon, versus a sensitivity of 1.5% from the 30-day time horizon.

## Discussion

In this work we explored the potential for developing a clinical decision support tool to identify children at high risk of an asthma exacerbation over a 30-, 60, and 180-day period. We used data that are commonly captured within EHR systems and also included spatial and temporal environmental data that are publicly available and not routinely captured within EHRs. A model that included all predictor variables had moderate performance (AUC ~ 76%) over all three time horizons, though the PPV was greatest for the 180-day period. Notably, model performance was predominately driven by data captured in the EHR, including patient demographics and service utilization history. The inclusion of spatial and temporal factors did not significantly improve model performance.

Prior studies have attempted to identify clusters of factors that can predict risk of asthma exacerbation in children and adolescents. The strongest predictive factor previously identified is having had an asthma exacerbation in the previous year [19–24]. Similarly, we also found that models that incorporated past healthcare utilization were most predictive. Other studies have incorporated laboratory values, such as aeroallergen sensitization and eosinophil and IgE levels, to predict exacerbations, though we found that few children in our cohort had these measures available. The Seasonal Asthma Exacerbation Index (saEPI) used these variables along with lung function parameters and asthma medication information to predict which children were at risk of exacerbation during the fall peak, and this index was shown to reliably predict which children were unlikely to experience an exacerbation, but was less successful identifying children who were at risk of an exacerbation [25]. Similarly, a machine learning model that included 142 variables, including demographics, neighborhood characteristics, laboratory results, vital signs, diagnosis codes, medications, insurance, encounters, and past healthcare utilization, had a high negative predictive value for patients with asthma who would not have an emergency department visit or hospitalization in the following year; however, the PPV of the model was under 25% [26]. Other models have included additional types of data, including patient symptom reports and remote monitoring data. Finkelstein and Jeong used daily adult asthma patient reports of symptoms and medication use and tele-monitoring of in-home spirometry to predict asthma exacerbations within a 1-week window [27]. This approach resulted in a model with a strong PPV for acute exacerbations; however, the heavy reliance on patient-supplied data would likely be difficult to implement broadly and in younger patients.

We found that the inclusion of spatial and temporal information was of limited added value in predicting future asthma exacerbations. Previous work by us and others has shown minimal added predictive value of neighborhood information for risk prediction models [28, 29]. Moreover, while environmental factors can impact risk of asthma exacerbation [30, 31], the epidemiological literature has shown relatively weak and inconsistent effects [32–34]. Importantly, most studies to date, including ours, have not included indoor environmental exposures, which may influence risk of asthma exacerbation. Moreover, readily available measures of outdoor environmental exposures may not be sufficiently granular to be informative on an individual patient level. For example, the data used in our model is derived from a single sensor within Durham County; thus, these data may not have sufficient resolution to provide an accurate estimate of exposure for all patients.

This work also highlights the impact of considering the time horizon over which predictions are made. Most of the studies conducted testing predictive models based on multiple patient-level factors have focused on prediction of exacerbations 6 to12 months into the future [19–24, 26]. Our models had slightly better performance for near-term (30-days) versus longer-term (180 days) outcomes. These results are in alignment with previous work showing that the granular nature of EHR data are well suited for nearer-term prediction [35]. However, when, we considered the performance of a decision rule with specified risk levels, the longer-term model had more practical real-world performance based on positive predictive value. The improved performance over a longer time horizon is due to the meaningfully higher event rate of events during the 180-day time horizon (7.3% vs 1.5%). While this result is not surprising, it highlights the importance of considering event rates when translating a risk model into a decision support tool.

In considering how a CDS tool to identify children at risk of an asthma exacerbation could be implemented, it is important to consider the types of data that are required for the underlying model and the clinical goals of the tool. Our results demonstrate that a model using data that are commonly available in our EHR system performed as well a model that includes basic spatio-temporal environmental data. Second, our data suggest that a CDS tool focused on relatively short-term outcomes would be most likely to provide actionable results. Further, the time horizon in which the model performs best informs the types of interventions that would be directed by the CDS tool.

There are some limitations to this work. Mainly, this is a single center study, and the results may not be fully generalizable. Additionally, model performance may be influenced by study location, wherein locales with different types of spatio-temporal variability would yield different results. For example, Durham County has relatively few poor air quality days, leading to a model that is less reliant on air pollution data. In contrast, locations such as Los Angeles or Atlanta tend to have more days with poor air quality; thus, environmental data may be more important for predictive models for patients living in those regions. Moreover, the outdoor environmental data used for this study was derived from a single sensor site in central Durham County; thus, these data may lack sufficient granularity to detect differences in exposures across the study cohort. Finally, we were not able to account for all variables that may have a significant impact on the likelihood of asthma exacerbations, including medication refill data, indoor environmental and direct respiratory virus exposures. Future studies will be needed to evaluate the importance of variables that could not be included in the current study and to evaluate the transportability of the models developed in this study to patient populations from other health systems.

In conclusion, we developed multiple predictive models for pediatric asthma exacerbations that included data that are commonly available in EHR systems as well as contextualizing spatio-temporal data. We found that the inclusion of spatio-temporal data did not significantly increase the performance of a model that used EHR data. Importantly, while our models exhibited nominally better performance over a 30-day time horizon compared to longer time periods, the decision rule metrics—based on sensitivity and PPV—were better for longer term (i.e., 180 day) time horizons. These findings have important implications for the design and implementation of CDS tools to identify children who would benefit from interventions to prevent asthma exacerbations.

## Supplementary Information

**Additional file 1: Table S1.** Diagnostic codes for asthma and atopic diseases. **Table S2.** Medication definitions. **Table S3.** Model Variables.

## Data Availability

The datasets generated and analyzed during the current study are not publicly available due to the need to protect patient privacy; however, de-identified analytic datasets are available from the corresponding author on reasonable request.

## Abbreviations

HER: Electronic health record
CDS: Clinical decision support
AUC: Area under the curve

## References

[1] Centers for Disease Control and Prevention. Most Recent National Asthma Data. https://www.cdc.gov/asthma/most_recent_national_asthma_data.htm. Accessed 23 Jul 2021.

[2] Centers for Disease Control and Prevention, National Center for Environmental Health. AsthmaStats: Asthma Attacks among People with Current Asthma, 2014–2017.

[3] QuickStats:Percentage* of All Emergency Department (ED) Visits Made by Patients with Asthma, by Sex and Age Group—National Hospital Ambulatory Medical Care Survey, United States 2014–2015. MMWR Morb Mortal Wkly Rep. 2018;67:167. 10.15585/mmwr.mm6705a5 PMC5812471 29420461

[4] Hogan AH, Carroll CL, Iverson MG, Hollenbach JP, Philips K, Saar K (2021). Risk factors for pediatric asthma readmissions: a systematic review. J Pediatr.

[5] Anise A, Hasnain-Wynia R (2016). Patient-centered outcomes research to improve asthma outcomes. J Allergy Clin Immunol.

[6] Goldstein BA, Navar AM, Pencina MJ, Ioannidis JPA (2017). Opportunities and challenges in developing risk prediction models with electronic health records data: a systematic review. J Am Med Inform Assoc.

[7] Stolte A, Merli MG, Hurst JH, Liu Y, Wood CT, Goldstein BA (2022). Using Electronic Health Records to understand the population of local children captured in a large health system in Durham County, NC, USA, and implications for population health research. Soc Sci Med.

[8] Hurst JH, Liu Y, Maxson PJ, Permar SR, Boulware LE, Goldstein BA (2020). Development of an electronic health records datamart to support clinical and population health research. J Clin Transl Sci.

[9] Tang M, Goldstein BA, He J, Hurst JH, Lang JE (2020). Performance of a computable phenotype for pediatric asthma using the problem list. Ann Allergy Asthma Immunol.

[10] Lang JE, Tang M, Zhao C, Hurst J, Wu A, Goldstein BA (2020). Well-child care attendance and risk of asthma exacerbations. Pediatrics.

[11] Bonito A, Bann C, Eicheldinger C, Carpenter L. Creation of new race-ethnicity codes and socioeconomic status (SES) indicators for medicare beneficiaries. http://www.ahrq.gov/research/findings/final-reports/medicareindicators/index.html. Accessed 9 Aug 2013.

[12] He J, Ghorveh MG, Hurst JH, Tang M, Alhanti B, Lang JE (2020). Evaluation of associations between asthma exacerbations and distance to roadways using geocoded electronic health records data. BMC Public Health.

[13] Friedman J, Hastie T, Tibshirani R (2010). Regularization paths for generalized linear models via coordinate descent. J Stat Softw.

[14] ranger: a fast implementation of random forests for high dimensional data in C++ and R | Journal of Statistical Software. https://www.jstatsoft.org/article/view/v077i01. Accessed 27 Feb 2022.

[15] Greenwell B, Boehmke B, Cunningham J. gbm: generalized boosted regression models. R package version. 2019;2(5).

[16] ROCR: visualizing classifier performance in R | Bioinformatics | Oxford Academic. https://academic.oup.com/bioinformatics/article/21/20/3940/202693. Accessed 27 Feb 2022. 10.1093/bioinformatics/bti623 16096348

[17] R Core Team. R: A language and environment for statistical computing. R Foundation for Statistical Computing. http://www.R-project.org/.

[18] Cohen HA, Blau H, Hoshen M, Batat E, Balicer RD (2014). Seasonality of asthma: a retrospective population study. Pediatrics.

[19] Haselkorn T, Zeiger RS, Chipps BE, Mink DR, Szefler SJ, Simons ER (2009). Recent asthma exacerbations predict future exacerbations in children with severe or difficult-to-treat asthma. J Allergy Clin Immunolo..

[20] Wu AC, Tantisira K, Li L, Schuemann B, Weiss ST, Fuhlbrigge AL (2011). Predictors of symptoms are different from predictors of severe exacerbations from asthma in children. Chest.

[21] Price DB, Rigazio A, Campbell JD, Bleecker ER, Corrigan CJ, Thomas M (2015). Blood eosinophil count and prospective annual asthma disease burden: a UK cohort study. Lancet Respir Med.

[22] Miller MK, Lee JH, Miller DP, Wenzel SE (2007). Recent asthma exacerbations: a key predictor of future exacerbations. Respir Med.

[23] Covar RA, Szefler SJ, Zeiger RS, Sorkness CA, Moss M, Mauger DT (2008). Factors associated with asthma exacerbations during a long-term clinical trial of controller medications in children. J Allergy Clin Immunol.

[24] Peters MC, Mauger D, Ross KR, Phillips B, Gaston B, Cardet JC (2020). Evidence for exacerbation-prone asthma and predictive biomarkers of exacerbation frequency. Am J Respir Crit Care Med.

[25] Hoch HE, Calatroni A, West JB, Liu AH, Gergen PJ, Gruchalla RS (2017). Can we predict fall asthma exacerbations? Validation of the seasonal asthma exacerbation index. J Allergy Clin Immunol.

[26] Luo G, He S, Stone BL, Nkoy FL, Johnson MD (2020). Developing a model to predict hospital encounters for asthma in asthmatic patients: secondary analysis. JMIR Med Inform.

[27] Finkelstein J, Jeong IC (2017). Machine learning approaches to personalize early prediction of asthma exacerbations: personalized prediction of asthma exacerbation. Ann NY Acad Sci.

[28] Bhavsar NA, Gao A, Phelan M, Pagidipati NJ, Goldstein BA (2018). Value of neighborhood socioeconomic status in predicting risk of outcomes in studies that use electronic health record data. JAMA Netw Open.

[29] Schuler A, O’Súilleabháin L, Rinetti-Vargas G, Kipnis P, Barreda F, Liu VX (2020). Assessment of value of neighborhood socioeconomic status in models that use electronic health record data to predict health care use rates and mortality. JAMA Netw Open.

[30] Stevens EL, Rosser F, Han Y-Y, Forno E, Acosta-Pérez E, Canino G (2020). Traffic-related air pollution, dust mite allergen, and childhood asthma in puerto ricans. Am J Respir Crit Care Med.

[31] Brandt SJ, Perez L, Künzli N, Lurmann F, McConnell R (2012). Costs of childhood asthma due to traffic-related pollution in two California communities. Eur Respir J.

[32] Rodriguez-Villamizar LA, Berney C, Villa-Roel C, Ospina MB, Osornio-Vargas A, Rowe BH (2016). The role of socioeconomic position as an effect-modifier of the association between outdoor air pollution and children’s asthma exacerbations: an equity-focused systematic review. Rev Environ Health.

[33] Zheng X, Ding H, Jiang L, Chen S, Zheng J, Qiu M (2015). Association between air pollutants and asthma emergency room visits and hospital admissions in time series studies: a systematic review and meta-analysis. PLoS ONE.

[34] Witonsky J, Abraham R, Toh J, Desai T, Shum M, Rosenstreich D (2019). The association of environmental, meteorological, and pollen count variables with asthma-related emergency department visits and hospitalizations in the Bronx. J Asthma.

[35] Goldstein BA, Pencina MJ, Montez-Rath ME, Winkelmayer WC (2017). Predicting mortality over different time horizons: which data elements are needed?. J Am Med Inform Assoc.

