# Supplementary material for: Environmental and clinical data utility in pediatric asthma exacerbation risk prediction models
Source: BMC Med Inform Decis Mak. 2022 Apr 22;22:108. doi: 10.1186/s12911-022-01847-0 (PMC9034565; doi:10.1186/s12911-022-01847-0)
Supplement: Supplementary file 1 — Additional file 1: Table S1. Diagnostic codes for asthma and atopic diseases. Table S2. Medication definitions. Table S3. Model Variables. [file 12911_2022_1847_MOESM1_ESM.docx]

**Supplemental Tables**

| **Supplemental Table 1.** Diagnostic codes for asthma and atopic diseases | | | |
| --- | --- | --- | --- |
| **Diagnosis** | | **ICD-9** | **ICD-10** |
| Asthma | | 493.* | J45.* |
| Atopic diseases | |  |  |
|  | Allergic rhinitis | 477.* | J30.* |
|  | Acute atopic conjunctivitis | 372.05 | H10.1* |
|  | Other chronic allergic conjunctivitis | 372.14 | H10.45 |
|  | Personal history of allergy, other than to medicinal agents, presenting hazards to health | V15.0* | Z91.0* |
|  | Anaphylactic reaction due to unspecified food | 995.6* | T78.* |
|  | Other adverse food reactions, not elsewhere classified | 995.7 | T78.* |
|  | Atopic dermatitis and related conditions | 691.* | L20.* |

| **Supplemental Table 2.** Medication definitions | |
| --- | --- |
| **Category** | **Medication** |
| Rescue medications | Albuterol Dry Powder Inhaler [ProAir] |
|  | Albuterol Inhalant Solution |
|  | Albuterol Metered Dose Inhaler [ProAir, Proventil, Ventolin] |
|  | Albuterol Oral Tablet |
|  | Albuterol/ipratropium bromide Inhalant Solution [DuoNeb] |
|  | Albuterol/ipratropium bromide Metered Dose Inhaler [Combivent] |
|  | Ipratropium bromide Inhalant Solution [Atrovent] |
|  | Ipratropium bromide Metered Dose Inhaler [Atrovent] |
|  | Levalbuterol Inhalant Solution [Xopenex] |
|  | Levalbuterol Metered Dose Inhaler [Xopenex] |
| ICS | Beclomethasone Dipropionate Metered Dose Inhaler [Qvar] |
|  | Budesonide Dry Powder Inhaler [Pulmicort] |
|  | Budesonide Inhalant Solution [Pulmicort] |
|  | Ciclesonide Metered Dose Inhaler [Alvesco] |
|  | Fluticasone furoate Dry Powder Inhaler [Arnuity Ellipta] |
|  | Fluticasone propionate Dry Powder Inhaler [Flovent] |
|  | Fluticasone propionate Metered Dose Inhaler [Flovent] |
|  | Mometasone furoate Dry Powder Inhaler [Asmanex] |
| LTRA | Montelukast Oral Tablet [Singulair] |
|  | Zileuton Oral Tablet [Zyflo] |
| LABA | Salmeterol inhalant product [Serevent] |
| LAMA | Tiotropium Metered Dose Inhaler [Spiriva] |
| Combination therapies | Budesonide / formoterol fumarate Metered Dose Inhaler [Symbicort] |
|  | Fluticasone furoate / vilanterol Dry Powder Inhaler [Breo] |
|  | Fluticasone propionate / salmeterol Metered Dose Inhaler [Advair] |
|  | Fluticasone propionate / salmeterol Dry Powder Inhaler [Advair] |
|  | Mometasone fumarate / formoterol furoate Metered Dose Inhaler [Dulera] |
| Biologics | Omalizumab [Xolair] |
|  | Mepolizumab [Nucala] |
| LTRA, leukotriene receptor antagonist; LABA, long-acting beta agonist; LAMA, long-acting muscarinic agent | |

| **Supplemental Table 3.** Model Variables | | | | | |
| --- | --- | --- | --- | --- | --- |
| Model | Overall Model | Temporal Factors | Clinical Factors | Spatial Factors | Parsimonious Model |
| Average temperature of the last day in the prior month | X | X |  |  |  |
| Log_10_ of the maximum wind speed in the last day of the prior month | X | X |  |  |  |
| Log_10_ of the number of positive influenza tests in the last day of the prior month | X | X |  |  |  |
| Average PM2.5 on the last day of the prior month | X | X |  |  |  |
| Log_10_ of the imputed tree pollen count on the last day of the prior month | X | X |  |  |  |
| Log_10_ of the imputed weed pollen count on the last day of the prior month | X | X |  |  |  |
| Log_10_ of the imputed grass pollen count on the last day of the prior month | X | X |  |  |  |
| Current month | X | X |  |  | X |
| Insurance status in the prior month (self-pay, private, public, unknown/other) | X |  | X |  |  |
| Patient sex (male, female) | X |  | X |  | X |
| Patient race (non-Hispanic white, non-Hispanic Black, Hispanic, Other, Unknown) | X |  | X |  | X |
| Patient age at current month | X |  | X |  | X |
| Patient BMI in the prior month | X |  | X |  | X |
| Presence of obesity diagnosis in the prior month | X |  | X |  |  |
| Presence of eczema diagnosis in the prior month | X |  | X |  | X |
| Presence of allergic rhinitis or conjunctivitis diagnosis in the prior month | X |  | X |  | X |
| Presence of food allergy diagnosis in the prior month | X |  | X |  | X |
| Active prescription for LTRA medication in the prior month | X |  | X |  | X |
| Active prescription for biologic medication in the prior month | X |  | X |  | X |
| Active prescription for ICS medication in the prior month | X |  | X |  | X |
| Any encounter in the past 30 days | X |  | X |  |  |
| Ambulatory visit encounter in the past 30 days | X |  | X |  |  |
| ED encounter in the past 30 days | X |  | X |  |  |
| ED-to-inpatient encounter in the past 30 days | X |  | X |  |  |
| Inpatient encounter in the past 30 days | X |  | X |  |  |
| Number of asthma exacerbations in the prior month | X |  | X |  |  |
| Number of hospital encounters more than 24 hours for asthma exacerbation in the prior month | X |  | X |  |  |
| Number of ED and hospital encounters less than 24 hours for asthma exacerbation in the prior month | X |  | X |  |  |
| Number of urgent care encounters for asthma exacerbation in the prior month | X |  | X |  |  |
| Number of outpatient encounters for asthma exacerbation in the prior month | X |  | X |  |  |
| Number of asthma exacerbations in the prior year | X |  | X |  |  |
| Number of hospital encounters more than 24 hours for asthma exacerbation in the prior year | X |  | X |  |  |
| Number of ED and hospital encounters less than 24 hours for asthma exacerbation in the prior year | X |  | X |  |  |
| Number of urgent care encounters for asthma exacerbation in the prior year | X |  | X |  |  |
| Number of outpatient encounters for asthma exacerbation in the prior year | X |  | X |  |  |
| Number of historical asthma exacerbations in Winter (December, January, February) | X |  | X |  |  |
| Number of historical asthma exacerbations in Spring (March, April, May) | X |  | X |  |  |
| Number of historical asthma exacerbations in Summer (June, July, August) | X |  | X |  |  |
| Number of historical asthma exacerbations in Fall (September, October, November) | X |  | X |  |  |
| Distance between home address to 55 MPH+ highway (miles) in the prior month | X |  |  | X |  |
| Distance between home address to 35 MPH+ highway (miles) in the prior month | X |  |  | X |  |
| Distance between home address to a public park (miles) in the prior month | X |  |  | X |  |
| SES scores by census tract for the patient’s address in the prior month | X |  |  | X |  |
| Tree cover per capita of the block the patient was living in in the prior month | X |  |  | X |  |
